# Supplementary material for: Polyketide synthase gene clusters in the ericoid mycorrhizal fungus Oidiodendron maius: genome-wide prediction, expression profiling, and functional characterization of knockout mutants
Source: Front Plant Sci. 2026 Sep 16;17:1924430. doi: 10.3389/fpls.2026.1924430 (PMC13623903; doi:10.3389/fpls.2026.1924430)
Supplement: Supplementary file 1 [file SupplementaryFile1.zip › Supplementary Materials/Supplementary Folder 1.zip!/Oidma1_AssemblyScaffolds_Repeatmasked/knownclusterblast/cluster37/jgi.p_Oidma1_148331_mibig_hits.html]

| MIBiG Protein | Description | MIBiG Cluster | MiBiG Product | % ID | % Coverage | BLAST Score | E-value |
| --- | --- | --- | --- | --- | --- | --- | --- |
| ADC45557.1 | isomerase | BGC0000093 | Meilingmycin\_biosynthetic\_gene\_cluster | 31 | 33.5907335907 | 149 | 2e-35 |
| ADC45548.1 | mandelate\_racemase | BGC0000093 | Meilingmycin\_biosynthetic\_gene\_cluster | 30 | 28.4749034749 | 101 | 4.8e-21 |
| BAE95548.1 | mandelate\_racemase/muconate\_lactonizing\_enzyme | BGC0000703 | Kanamycin\_biosynthetic\_gene\_cluster | 31 | 22.4903474903 | 86 | 2.1e-16 |
